# Vaccination coverage estimates and utilization patterns of inactivated enterovirus 71 vaccine post vaccine introduction in Ningbo, China

**DOI:** 10.1186/s12889-021-11198-6

**Published:** 2021-06-10

**Authors:** Lixia Ye, Jieping Chen, Ting Fang, Rui Ma, Jianmei Wang, Xingqiang Pan, Hongjun Dong, Guozhang Xu

**Affiliations:** Ningbo Municipal Center for Disease Prevention and Control, Institute of Immunization and Prevention, Yongfeng Road, Haishu District, Ningbo, 315010 China

**Keywords:** Inactivated enterovirus 71 vaccine, Coverage, Timeliness, Simultaneous administration

## Abstract

**Background:**

In China, enterovirus 71 (EV71) is the major etiological agents of hand foot mouth disease that poses severe risks to children’s health. Since 2015, three inactivated EV71 vaccines have been approved for use. Previous studies indicated the high willingness of EV71 vaccination in eastern China. However, few studies have assessed coverage and utilization patterns of EV71 vaccine in China.

**Methods:**

Children born during 2012–2018 were sampled and their records were abstracted from Ningbo childhood immunization information management system. Descriptive statistics characterized the study population and assessed coverage and timeliness for EV71 vaccination. Simultaneous administration patterns as well as type of EV71 vaccine used were also evaluated. Bivariate and multivariable analysis was used to examine the relationship of socio-demographic characteristics with vaccination coverage and timeliness.

**Results:**

Of 716,178 children living in Ningbo. One hundred seventy-two thousand two hundred thirty-six received EV71 vaccine with a coverage rate of 24.05% and only 8.61% received vaccination timely. 21.97% of children received the complete two dose EV71 series but only 6.49% completed timely. Vaccination coverage and timeliness increased significantly from 2012 birth cohort to 2018 birth cohort. Relatively higher coverage and timeliness were observed in resident children, Inner districts, high socioeconomic areas and large-scaled immunization clinics. Of 329,569 doses of EV71 vaccine, only 5853(1.78%) doses were administered at the same day as other vaccines.

**Conclusions:**

There is a need for increasing EV71 vaccination coverage and timeliness as well as eliminating disparities among different populations. Our study highlights the importance of simultaneous administration to increasing coverage and timeliness of EV71 vaccination.

## Introduction

Hand, foot and mouth disease(HFMD) has been widely considered as a highly infectious exanthematous illness mostly affecting children under 5 years old and has caused substantial burden in Asia [1–3], which is characterized with fever, cutaneous lesions on hands, feet, groin and buttocks, as well as painful oral lesions [4]. In China, a total of 13.7 million HFMD cases were reported nationwide during the period from 2008 through 2015 with the highest incidence of any infectious disease [5] and 123,261 cases and 3322 cases of them rapidly developed severe and life-threatening complications respectively [5], such as meningitis, encephalitis and pulmonary edema. Enterovirus 71(EV71) is one of the major etiological agents of HFMD [6, 7]. As EV71 is a neurotropic virus, EV71-associated HFMD tends to be more severe and even caused fatal neurological complications [6], accounting for 73.8% of severe cases and 92.5% of fatal cases [5].

To date, since there is no effective antiviral treatment for EV71 infection available yet [6, 7], EV71 vaccine has been considered as a promising preventive intervention against EV71-associated HFMD [7, 8]. Three inactivated EV71 vaccines have been licensed and commercially available in China since December 2015 [8], including two Vero cell-based vaccines developed by Sinovac Biotech(Beijing) [9] and Vigoo Biological(Beijing) [10] respectively and a whole-virus vaccine developed by the Institute of Medical Biology, Chinese Academy of Medical Scienses(CAMS) [11]. The vaccine efficacy of all three EV71 vaccines exceeded 90 and 98.8% against EV71-associated HFMD after one dose and two doses [9–11]. Therefore, 2 doses of EV71 vaccine, as a private-sector vaccine that is voluntarily and paid for out-of pocket by parents or caregivers, are recommended by Chinese Center for Disease Prevention and Control (China CDC) to children aged 6–59 months with an interval of month, especially to infants aged 6–12 months for best efficacy [12].

As achieving a high coverage of EV71 vaccine is necessary for reducing EV71-associated HFMD significantly [11, 13], vaccination coverage is considered to be a critical indicator of the EV71 vaccines’ performance on EV71-associated HFMD control. In the recent years, various studies about the EV71 vaccine acceptance [14–17] indicated that 68.79% of parents were willing to vaccinate their children with EV71 vaccines in the eastern China [14]. However, it is challenging to determine a proper timing for EV71 vaccine since at least 20 doses of National Immunization Programme(NIP) vaccines also need to be administered at the same age of EV71 vaccination in China, especially within first 2 years of life [18]. A recent study showed that simultaneous administration of combined EV71 vaccine with other NIP vaccines was as effective and safe as separate administration of EV71 vaccine [19], which raised the likelihood of simultaneous administration of combined EV71 vaccine with other vaccines in order to increase the vaccination uptake. Up to now, little is known about the coverage of EV71 vaccine as well as the utilization pattern in different population, including the simultaneous administration of combined EV71 vaccine with other vaccines. Although Guangdong and Fujian provinces have estimated the EV71 vaccination coverage [20, 21], these were only rough estimates using the reported coverage data from China immunization information system rather than the individual-level records of children which might result in biased estimates. Moreover, the utilization pattern of EV71 vaccine, such as simultaneous administration with other vaccines and the type of EV71 vaccine using in different age group, has not been reported till now.

Ningbo is an economically developed large coastal city located in the east coastline of China and has a population of approximately 6.08 million registered population and 4.80 million migrant population. As one of the earliest childhood electronic immunization registries in China, Ningbo Childhood Immunization Information Management System(NBCIIMS) now contains continuous, real-time and individual-level demographic information and vaccination records dating back to children born in 2000 and can provide a ideal tool for estimating EV71 vaccination coverage. EV71 vaccine has been available in Ningbo since November 2016. After vaccine introduction, a survey conducted by Ding K et al. [15] found that 70.94% of parents were willing to vaccinate their children with EV71 vaccine. In this study, we estimated the real-world coverage of EV71 vaccine as well as completeness and timeliness of vaccination in the children born in 2012–2018 using the data from NBCIIMS. Furthermore, we also characterized disparities in utilization of two kinds of EV71 vaccines(Vero cell-based vaccine vs. whole-virus vaccine) and evaluated simultaneous administration patterns of EV71 vaccination in Ningbo post vaccine introduction.

## Methods

### Data resources

A cross-sectional study was conducted in this study. The anonymized individual records of children were extracted from NBCIIMS on Dec 31st, 2019. NBCIIMS is a computerized, population-based, real-time, children immunization registration system that has been used in all immunization clinics under the authority of Ningbo Municipal CDC since 2005. At birth or moving to Ningbo for more than 3 months, children under 7 years old are registered in NBCIIMS with a unique identification number by the immunization clinics in Ningbo. Therefore, all the children in Ningbo are included in NBCIIMS, even if they don’t receive any vaccine. For each child, historical and current vaccination records should be registered into NBCIIMS and updated in real time(including vaccination in Ningbo and the historical vaccination at the clinics out of Ningbo), which provides an ideal tool to make an accurate estimate of the vaccination coverage.

According to *the Technical guidelines for the use of inactivated enterovirus 71 vaccines(2016),* [12] and Products instructions of EV71 vaccines, a 2-dose EV71 vaccination series with 1 month interval is recommended. Vero cell-based EV71 vaccines are recommended to children aged 6–35 months while whole-virus EV71 vaccine is recommended to children aged 6–59 months. For best efficacy, 2-dose EV71 vaccination series should been completed at 6–12 months of age. Therefore, we included the children born from Jan 1, 2012 to Dec 31, 2018 and registered in NBCIIMS as our target population in this study. We excluded the children 1) who visited the clinic and received the vaccination temporarily during their stay in Ningbo; 2) designated as “permanently inactive” (i.e. deceased) or “moved elsewhere” by Dec 31st 2019.

### Variable definitions and data collection

Individual-level records were extracted from NBCIIMS, including sex, birthdate, immigration status, district of residence, the scale of registered immunization clinic and dates of all the vaccination regardless of whether the vaccine was received in Ningbo or out of Ningbo. For all the EV71 vaccination received in Ningbo, the type of EV71 vaccine was also extracted.

Immigration status was categorized as three types: resident children, migrant children from other municipalities and foreign children. As for district of residence, urbanicity was grouped into two categories: 1) Inner districts included the urban districts of Haishu, Jiangbei, Yinzhou, Zhenhai, Beilun and Fenghua; 2) Outer districts included the rural districts of Yuyao, Cixi, Ninghai and Xiangshan.

According to *2018 statistics from Ningbo municipal bureau of statistics*, we also classified the socioeconomic level of resident areas into two categories by Gross Domestic Product (GDP) per capita: Beilun, Zhenhai, Jiangbei and Yinzhou were classified as high level for GDP per capita > GDP per capita of Ningbo city (20,038 USD) and other areas were classified as low level for GDP per capita <GDP per capita of Ningbo city (20,038 USD). As for the factor about the immunization clinic, the scale of immunization clinics were divided into three strata by the number of children registered in the immunization clinic (large-scaled for ≥10,000 registered children; midsized for 5000 ~ 9999 registered children; small-sized for less than 5000 registered children).

The vaccination coverage rate was defined as the proportion of vaccinated children(≥1dose) among the target population. As to on-time receipt, Children were considered “on-time” if they received at least 1dose of EV71 vaccine during 6–12 months of age. Series completion rate was defined as the proportion of children who received 2 doses of EV71 vaccines among the target population while on-time series completion was considered as “on-time” if children complete 2 doses of EV71 vaccines during 6–12 months of age. Vaccination was regarded invalid if it 1) was administrated before 6 months of age or 2) was administrated < 28 days from the previous dose of EV71 vaccine. Simultaneous administration of EV71 vaccine was defined as administering EV71 vaccination with other vaccines at different anatomic sites by separate syringe in the same visit day.

### Statistical analysis

Descriptive statistics were calculated for each demographic characteristics and vaccination outcome, including counts and proportions. Coverage rate, on-time receipt, series completion and on-time series completion of EV71 vaccine were calculated by demographic characteristics. To gain a complete picture of how demographic characteristics predicted vaccination status, multivariate logistic regressions were used to analyze the associations between the demographic characteristics and vaccination/on-time receipt at significance level *p* < 0.05 among children born in 2016–2018 since EV71 vaccine was licensed after Dec 2015. Adjusted Odds ratio(aOR) and 95% confidence intervals(CIs) were calculated. The final model included sex, birth year, immigration status and urbanicity, the socioeconomic level of resident areas and the scale of immunization clinics. The cumulative proportion of children vaccinated over time by birth cohort was calculated. The proportion of simultaneous administration of EV71 vaccine among all vaccinated children was described by different demographic characteristics and evaluated using Wald chi-square test at significance level *p* < 0.05. In addition, among all the EV71 vaccination received in Ningbo, the proportions of two types of EV71 vaccines used were also estimated.

## Results

A total of 716,178 eligible children were included in this study. Table 1 showed the study population characteristics. The study population consisted of more males (52.93%), more children from inner districts(55.40%) and greater numbers of children from low-level socio-economic areas. 53.11% were migrant children while 46.79% were resident children and 0.10% were foreign children. The subjects born during 2012–2018 ranged from 13.30 to 15.57%. 43.79% of the subjects were registered in the large-scale immunization clinics while 25.24 and 30.97% of the subjects were registered in the midsized and small-sized immunization clinics.

**Table 1:** Distribution of characteristics of the study population, children born during 2012–2018 in Ningbo, China

Demographics	No.	Frequency(%)
Total	716,178	100.00
Sex		
Male	379,102	52.93
Female	337,076	47.07
Immigration status		
Resident children	335,120	46.79
Migrant children	380,338	53.11
Foreign children	720	0.10
Urbanicity		
Inner	396,782	55.40
Outer	319,396	44.60
Birth year		
2012	102,425	14.30
2013	95,979	13.40
2014	107,674	15.03
2015	97,433	13.60
2016	111,492	15.57
2017	105,894	14.79
2018	95,281	13.30
Socioeconomic level of resident areas		
High	258,474	36.09
Low	457,704	63.91
Scale of immunization clinics		
Large-scaled	313,599	43.79
Midsized	180,796	25.24
Small-sized	221,783	30.97

### Vaccination coverage and timeliness of EV71 vaccine

#### Vaccination coverage of EV71 vaccine

Of the 716,178 subjects, 172,236 received at least 1 dose of EV71 vaccine, for a vaccination coverage rate of 24.05%(95%CI: 23.95–24.15%). Vaccination coverage of EV71 varied substantially by birth cohorts(Table 2), with the lowest coverage reported among those born in 2012(0.49, 95%CI: 0.45–0.53%) and the highest coverage reported among those born in 2017(48.00, 95%CI: 47.70–48.30%). By immigrant status, the EV71 vaccine coverage rate among resident children, migrant children and foreign children were 26.24, 22.13 and 19.31% respectively. In addition, the vaccination coverage were significantly higher among children living in inner districts(27.97, 95%CI: 27.83–28.11%) than outer districts(19.18, 95%CI: 19.04–19.31%), in high socioeconomic area(29.62, 95%CI: 29.44–29.79%) than low socioeconomic area(20.90, 95%CI: 21.79–21.02%). As for the factor of immunization clinics, children were slightly less likely to get vaccinated with EV71 vaccine in the small-sized clinics (Table 2). The multivariate logistic regression analysis showed that the uptake of EV71 vaccine was associated with resident children, inner districts, increasing of birth year, high socioeconomic level of resident area and midsized immunization clinics positively (Table 3).

**Table 2:** EV71 vaccination coverage among children born during 2012–2018 in Ningbo, China

Characteristics	EV71 vaccination coverage(≥1dose)		EV71 vaccination coverage on-time^a^	
	No.	rate %(95%CI)	No.	rate %(95%CI)
Total	172,236	24.05(23.95–24.15)	61,661	8.61(8.54–8.67)
Sex				
Male	90,124	23.77(23.64–23.91)	32,081	8.46(8.37–8.55)
Female	82,112	24.36(24.22–24.51)	29,580	8.78(8.68–8.87)
Immigration status				
Resident children	87,935	26.24(26.09–26.39)	33,512	10.00(9.90–10.10)
Migrant children	84,162	22.13(22.00–22.26)	28,095	7.39(7.30–7.47)
Foreign children	139	19.31(16.48–22.38)	54	7.50(5.68–9.67)
Urbanicity				
Inner	110,989	27.97(27.83–28.11)	44,136	11.12(11.03–11.22)
Outer	61,247	19.18(19.04–19.31)	17,525	5.49(5.41–5.57)
Birth year				
2012	501	0.49(0.45–0.53)	N/A	N/A
2013	2675	2.79(2.68–2.89)	N/A	N/A
2014	10,822	10.05(9.87–10.23)	N/A	N/A
2015	22,590	23.19(22.92–23.45)	338	0.35(0.31–0.39)
2016	42,677	38.28(37.99–38.56)	10,316	9.25(9.08–9.42)
2017	50,826	48.00(47.70–48.30)	21,998	20.77(20.53–21.02)
2018	42,145	44.23(43.92–44.55)	29,009	30.45(30.15–30.74)
Socioeconomic level of resident areas				
High	76,556	29.62(29.44–29.79)	33,244	12.86(12.73–12.99)
Low	95,680	20.90(20.79–21.02)	28,417	6.21(6.14–6.28)
Scale of immunization clinics				
Large-scaled	76,687	24.45(24.30–24.60)	31,915	10.18(10.07–10.28)
Midsized	43,279	23.94(23.74–24.14)	12,280	6.79(6.68–6.91)
Small-sized	52,270	23.57(23.39–23.75)	17,466	7.88(7.76–7.98)

^a^EV71 vaccination on-time was defined as receiving at least 1 dose of EV71 vaccine during 6-12 months of age

**Table 3:** Factors associated with receipt of EV71 vaccination(≥1dose) by demographic characteristics for children born during 2016–2018 in Ningbo, China

Characteristics	EV71 vaccination (≥1dose)		EV71 vaccination on-time^a^	
	aOR(95%CI)	*P*	aOR(95%CI)	*P*
Sex				
Male	1.00(0.98–1.01)	0.082	0.99(0.97–1.01)	0.176
Female	Reference		Reference	
Immigration status				
Resident children	1.48(1.15–1.90)	0.002	1.41(1.03–1.92)	0.032
Migrant children	1.08(0.84–1.39)	0.529	0.98(0.72–1.34)	0.895
Foreign children	Reference		Reference	
Urbanicity				
Inner	1.48(1.45–1.50)	< 0.001	1.48(1.44–1.52)	< 0.001
Outer	Reference		Reference	
Birth year				
2018	1.30(1.28–1.33)	< 0.001	4.63(4.52–4.75)	< 0.001
2017	1.52(1.49–1.54)	< 0.001	2.69(2.62–2.76)	< 0.001
2016	Reference		Reference	
Socioeconomic level of resident areas				
High	1.45(1.42–1.48)	< 0.001	1.95(1.90–2.00)	< 0.001
Low	Reference		Reference	
Scale of immunization clinics				
Large-scaled	1.01(1.00–1.03)	0.142	1.25(1.22–1.27)	< 0.001
Midsized	1.07(1.05–1.10)	< 0.001	0.82(0.80–0.85)	< 0.001
Small-sized	Reference		Reference	

*aOR* Adjusted odds ratio, *CI* Confidence interval

^a^EV71 vaccination on-time was defined as receiving at least 1 dose of EV71 vaccine during 6-12 months of age

#### Timeliness of EV71 vaccination

Timeliness of EV71 vaccination before 12 months of age was also evaluated in our study (Table 2). Only 61,661(8.61%) children received EV71 vaccination on time(95%CI: 8.54–8.67%). Among children born in 2015–2018, as the birth year increased, on-time coverage rate increased sharply, from lowest on-time coverage among those born in 2015(0.35, 95%CI: 0.31–0.39%) to the highest on-time coverage among those born in 2018(30.45, 95%CI: 30.15–30.74%), the most recent year in which the entire cohort was old enough to receive EV71 vaccination on time. Similar with EV71 vaccination coverage, higher on-time coverage rates of EV71 vaccine were observed in resident children(10.00, 95%CI: 9.90–10.10%), children living in inner districts(11.12, 95%CI: 11.03–11.22%) and high socioeconomic areas(12.86, 95%CI: 12.73–12.99%). By the scale of the immunization clinics, 10.18%(95%CI: 10.07–10.28%) of children registered in large-scaled clinics get vaccinated with EV71 on time while only 6.79%(95%CI: 6.68–6.91%) of children registered in midsized clinics were vaccinated on time which was least. In the multivariate logistic regression analysis, factors associated with increased likelihood of on-time receipt of EV71 vaccine included resident children, inner districts, increasing of birth year, high socioeconomic level of resident area, registered in large-scale immunization clinics. Registered in midsized immunization clinics were associated with decreased likelihood of on-time receipt of EV71 vaccine (Table 3).

### Series completion and timeliness

As shown in Table 4, 157,333 children received the complete two dose EV71 series, for a series completion rate of 21.97%(95%CI: 21.87–22.06%). However, only 6.49%(95%CI: 6.43–6.54%) of children received a complete series on time. Across the birth cohort from 2012 to 2018, the EV71 series completeness increased gradually over years and reached the highest rate(45.17, 95%CI: 44.87–45.47%) among those born in 2017 while on-time series completion rate was highest(23.58, 95%CI: 23.31–23.85%) among those born in 2018. The substantial differences were also observed between inner and outer districts. Children from inner districts were more likely to complete the two dose EV71 series(25.86, 95%CI: 25.73–24.00%) compared with children from outer districts(17.13, 95%CI: 17.00–17.26%), and on-time series completion rate was 8.52%(95%CI: 8.43–8.61%) among children from inner districts which was more than 2 times of the rate among those from outer districts(Table 4). By the socioeconomic level of resident areas, 27.52%(95%CI: 27.35–27.70%) of children from high socioeconomic areas received a complete series and 9.84%(95%CI: 9.73–9.96%) completed on time. In the contract, only 18.83%(95%CI: 18.72–18.94%) of those from low socioeconomic areas received a complete series and 4.59%(95%CI: 4.53–4.65%) completed on time. By the immigration status, the series completion rate and on-time rate of resident children were 24.69%(95%CI: 24.54–24.83%) and 8.04%(95%CI: 7.94–8.13%) respectively, which were highest. Although the series completion rate among children registered in midsized clinics(21.65, 95%CI: 21.46–21.84%) was only slightly higher than small-sized clinics, the on-time series completion rates were lowest in midsized clinics(4.98, 95%CI: 4.88–5.08%) and highest in large-scaled clinics(7.78, 95%CI: 7.69–7.88%).

**Table 4:** Two-doses EV71 vaccine series completion status among children born during 2012–2018 in Ningbo, China

Characteristics	Series completed		Series completed on-time^a^	
	No.	rate %(95%CI)	No.	rate %(95%CI)
Total	157,333	21.97(21.87–22.06)	46,448	6.49(6.43–6.54)
Sex				
Male	82,246	21.69(21.56–21.83)	23,996	6.33(6.25–6.41)
Female	75,087	22.28(22.14–22.42)	22,452	6.66(6.58–6.75)
Immigration status				
Resident children	82,735	24.69(24.54–24.83)	26,928	8.04(7.94–8.13)
Migrant children	74,472	19.58(19.45–19.71)	19,482	5.12(5.05–5.19)
Foreign children	126	17.50(14.79–20.48)	38	5.28(3.76–7.17)
Urbanicity				
Inner	102,624	25.86(25.73–26.00)	33,808	8.52(8.43–8.61)
Outer	54,709	17.13(17.00–17.26)	12,640	3.96(3.89–4.03)
Birth year				
2012	386	0.38(0.34–0.42)	N/A	N/A
2013	2223	2.32(2.22–2.41)	N/A	N/A
2014	9560	8.88(8.71–9.05)	N/A	N/A
2015	20,661	21.21(20.95–21.46)	108	0.11(0.09–0.13)
2016	39,717	35.62(35.34–35.91)	7437	6.67(6.52–6.82)
2017	47,836	45.17(44.87–45.47)	16,438	15.52(15.31–15.74)
2018	36,950	38.78(38.47–39.09)	22,465	23.58(23.31–23.85)
Socioeconomic level of resident areas				
High	71,143	27.52(27.35–27.70)	25,438	9.84(9.73–9.96)
Low	86,190	18.83(18.72–18.94)	21,010	4.59(4.53–4.65)
Scale of immunization clinics				
Large-scaled	70,339	22.43(22.28–22.58)	24,404	7.78(7.69–7.88)
Midsized	39,142	21.65(21.46–21.84)	8998	4.98(4.88–5.08)
Small-sized	47,852	21.58(21.41–21.75)	13,046	5.88(5.78–5.98)

^a^Series completed on-time was defined as completing 2-dose of EV71 vaccination during 6-12 months of age

### Age at immunization and cumulative coverage of EV71 vaccine

**Fig. 1:**
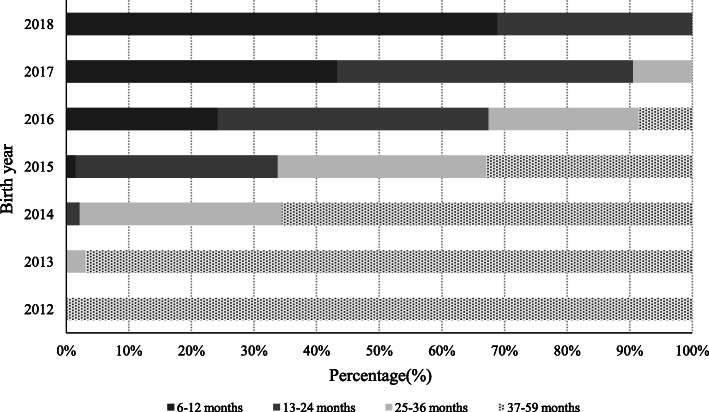
Age distribution of first dose EV71 vaccination of the children born during 2012 to 2018 in Ningbo

Among 172,236 children who vaccinated with EV71 vaccine, most children received first dose at age of 6–24 months(35.80% for 6–12 months of age and 36.67% for 13–24 months of age) and only 27.53% of children received first dose after 2 years of age. Figure 1 illustrated the distribution of vaccination age for first dose of EV71 vaccine by birth year. The proportion of children who were vaccinated at 6–12 months by birth cohort were 0.00, 0.00, 0.00, 1.49, 24.17, 43.28 and 68.83% for each of the 2012 through 2018 birth cohorts respectively, which showed a positive growth trend by birth cohort(*P* < 0.001).

**Fig. 2:**
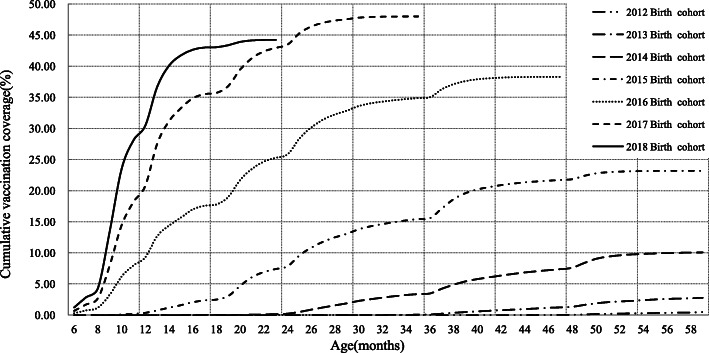
Cumulative vaccination coverage of EV71 vaccine among children born during 2012 to 2018 in Ningbo

The plot of cumulative vaccination coverage showed that the percent of children who received EV71 vaccination prior to 18 months of age increased significantly for children born in 2015–2018(*P* < 0.001). At 18 months of age, the vaccine coverage rates were 2.49, 17.76, 35.71 and 43.06% among children born in 2015, 2016, 2017 and 2018 respectively. For children born in 2012–2014 who were more than 2 years old when the EV71 vaccines were approved for use in China, the vaccination coverage of EV71 was consistently low(Fig. 2).

### Type of EV71 vaccine

**Fig. 3:**
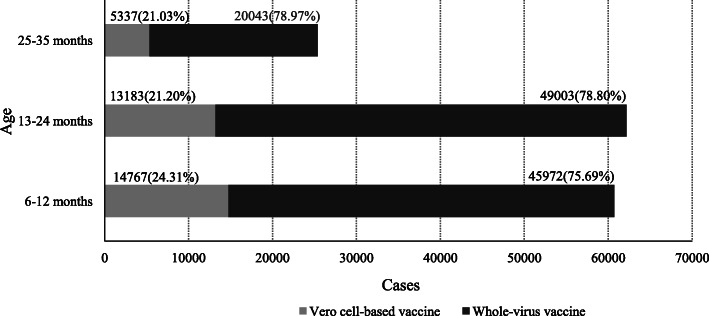
EV71 vaccine types using among the vaccinated children aged 6–35 months in Ningbo(*N* = 148,305)

Of 148,305 children who received EV71 vaccine at the age of 6–35 months, 33,287(22.44%) chose Vero cell-based EV71 vaccine;115,018(77.56%) chose whole-virus EV71 vaccine. There was a significant difference in the distribution of EV71 vaccine type by age (*P* < 0.001). 24.31% received Vero cell-based EV71 vaccine among the children aged 6–12 months which was higher than children over 12 months old(Fig. 3).

### Simultaneous administration of EV71 vaccine

A few children had EV71 simultaneously administered with other vaccines. Including all 329,569 doses of EV71 vaccine, only 5853(1.78%) doses administrations occurred at the same day as other vaccines. 5712(1.73%) doses were administered with another vaccine while 141(9.04%) doses were administered with two or more other vaccines (Table 5). There was significant difference in simultaneous administration by the doses of EV71 vaccine. 2.30% of children received EV71 vaccine with other vaccines at the first dose while the proportion of simultaneous administration reduced to only 1.21% at the second dose. Moreover, as shown in Table 5, simultaneous administration was more likely occurred in migrant children(2.81%), inner districts(1.84%), children aged 6–12 months(2.39%), high socioeconomic areas(2.05%), large(1.99%) and midsized(1.94%) immunization clinics.

**Table 5:** Simultaneous administration of EV71 vaccine among children born during 2012–2018 in Ningbo, China

Characteristics	Total, N	EV71 vaccination alone, N(%)	Simultaneous administration of EV71 vaccine, N(%)		χ^2b^	*P*^*b*^
			with another vaccine	with two or more vaccines		
Total	329,569	323,716(98.22)	5712(1.73)	141(0.04)		
Dose of EV71						
1st dose	172,236	168,283(97.70)	3850(2.24)	103(0.06)	559.19	< 0.001
2nd dose	157,333	155,433(98.79)	1862(1.18)	38(0.02)		
Sex						
Male	172,370	169,290(98.21)	3006(1.74)	74(0.04)	0.25	0.884
Female	157,199	154,426(98.24)	2706(1.72)	67(0.04)		
Immigration status						
Resident children	170,670	169,283(99.19)	1368(0.80)	19(0.01)	1795.43	< 0.001
Migrant children^a^	158,899	154,433(97.19)	4344(2.73)	122(0.08)		
Urbanicity						
Inner	213,613	209,686(98.16)	3840(1.80)	87(0.04)	15.40	< 0.001
Outer	115,956	114,030(98.34)	1872(1.61)	54(0.05)		
Age of vaccination						
6–12 months	119,754	116,886(97.61)	2804(2.34)	64(0.05)	444.21	< 0.001
13–24 months	121,004	119,158(98.47)	1806(1.49)	40(0.03)		
25–36 months	49,547	48,872(98.64)	661(1.33)	14(0.03)		
37–59 months	39,261	38,797(98.82)	441(1.12)	23(0.06)		
Socioeconomic level of resident areas						
High	147,699	144,676(97.95)	2964(2.01)	59(0.04)	118.11	< 0.001
Low	181,870	179,040(98.44)	2748(1.51)	82(0.05)		
Scale of immunization clinics						
Large-scaled	147,026	144,105(98.01)	2868(1.95)	53(0.04)	178.75	< 0.001
Midsized	82,421	80,820(98.06)	1545(1.87)	56(0.07)		
Small-sized	100,122	98,791(98.67)	1299(1.30)	32(0.03)		

^a^Migrant children included the children from other cities of china and foreign countries

^b^Comparison of proportion of simultaneous administration in the population with different demographic characteristics was evaluated by Chi-square test

Vaccines given simultaneously with EV71 vaccine included rotavirus(23.18%), meningococcal(17.10%), varicella(9.38%), JEV(8.27%), Hib(7.67%), Measles containing(6.59%), influenza(6.27%), PV(5.33%), DTP(4.22%), hepatitis B(4.00%), hepatitis A(3.90%), cholera(2.67%), pneumococcal(2.22%), mumps(0.73%), Hib-MenAC(0.73%), combined hepatitis A and B(0.15%), BCG(0.10%), DTP-Hib(0.09%) and DTP-IPV-Hib(0.03%) vaccines.

## Discussion

This study reported the real-world estimates of EV71 vaccination coverage in the children born in 2012–2018. In a large sample of children from Ningbo city which is located in economically-advanced eastern China, we found that the overall EV71 vaccination coverage of children was low, even though it was higher than those estimated for coverage reported in Guangdong [21] and Fujian province [20]. Nevertheless, high coverage is important because it can not only protect the individuals vaccinated but also create the additional benefits to society if sufficient herd immunity develops which happens when the vaccination coverage rate reached 80–90% [22, 23]. Outbreaks and prevalence of disease would occur as a result of the vaccination coverage falling down [22–25]. Thus, coverage of EV71 vaccination is an indicator for performance of EV71 vaccination among large population. In stark contrast to NIP vaccines with the sustained high coverages which were over 95% [18], the EV71 vaccination coverage in Ningbo was far below 80–90%. The discrepancy in coverage was likely due to the use of EV71 vaccine as a private-sector vaccine that needs to be paid out of pocket. Furthermore, as shown in previous studies [26–29], lack of information about EV71 vaccination was another reason leading to vaccine hesitancy. Parents needed to be fully informed to make vaccine-related decisions [30] and believed that health care workers (HCWs) were authoritative sources of information about vaccination and would make proper recommendation if necessary [31]. Unfortunately, most HCWs working in immunization clinics in Ningbo worked overload [32]. That made HCWs too busy to give parents the proper information, like effectiveness and side-effects of vaccine, to help parents judge whether receive vaccination or not. In contract, NIP vaccines are mandatory and free to the public. China has made great strides to maintain a high level of NIP vaccines coverage in the last few decades, including reminder-recall and standing orders for the children who missed vaccinations [18].

It was worth noting that the EV71 vaccination coverage was significantly higher in later than in earlier birth cohort. Since the EV71 vaccines were approved for use in Ningbo after 2016, earlier birth cohorts were unable to receive this vaccine at younger ages and the EV71 vaccination coverage of 2012–2014 birth cohorts remained quite low. On one hand, this may be due to the decreased visits to the immunization clinics after 2 years of age [18] and lack of information about EV71 vaccines; on the other hand, the parents of children over 2 years old were more reluctant to vaccinate their children with EV71 vaccine than the parents of children under 2 years old [15]. Among the 2015–2018 birth cohorts, the coverage and completeness of EV71 vaccination was increased significantly. This may partially be a result of vaccination education intervention program for new parents since 2016 in Ningbo, including issues on the importance of vaccination, the schedule of vaccination and immunization policy in China. Interestingly, although the EV71 vaccination coverage increased rapidly, there has still been a huge gap between vaccination intention and actual coverage. A previous study conducted by Ding K et al. in 2017 [15] indicated that 70.94% of parents were willing to vaccinated their children with EV71 vaccine in Ningbo but the actual coverage was only 24.05%. As a new vaccine licensed in 2015, Ding K et al’s study [15] showed that concerns about the potential side-effects of the EV71 vaccine were the main reason for the unwillingness of vaccination. Among the parents with EV71 vaccination intention, still 72.04% parents worried about the potential side-effects [15]. That might also be the barrier between vaccination intention and actual uptake.

Vaccination delays and dropouts was another problem of EV71 vaccination. Since the incidence rates of hand, foot and mouth disease in China peaked at 1 year of age and declined with age [5], 2-dose EV71 vaccination series at 6–12 months of age were recommended China CDC for best efficacy [12]. Therefore, timeliness of EV71 vaccination was considered to be another indicator of the effectiveness of EV71 vaccine. Actually, our study showed that only 6.49% of children completed 2 doses of EV71 vaccination on-time. Among the children vaccinated, 35.80% received first dose at 6–12 months of age. Even among 2018 birth cohort with the highest EV71 vaccine series completion rate, only 23.58% of children received 2 doses vaccination. According to the schedule of NIP vaccination, children have a very busy schedule of vaccination at 6–12 months of age that they need to finish nearly one-third of NIP vaccinations(6 doses) during this period of time. Furthermore, simultaneous administration of EV71 vaccine was not recommended by China CDC due to lack of data about concomitant administration [12]. Therefore, many parents had to delay the EV71 vaccination or even let their children remain undervaccinated. In our study, just 1.78% of parents chose to simultaneously administer EV71 vaccination with other vaccines and simultaneous administration was more likely occurred among the children aged 6–12 months. We also found that rotavirus and meningococcal vaccines which were given at 6–12 months of age were the most usual vaccines administered with EV71 vaccine. A recent study [19] showed that simultaneous administration of combined EV71 vaccine with Hepatitis B and group A meningococcal vaccine was as effective and safe as separate administration of EV71 vaccine, suggesting that simultaneous administration could be a reliable alternative as well as good supplementary to conventional planned vaccination schedule. In order to improve the coverage and timeliness of EV71 vaccination, simultaneous administration strategy for EV71 vaccine should be redesigned and add to the current guidelines.

Consistent with previous studies [18, 33, 34], we found meaningful disparities in coverage and timeliness of EV71 vaccination by children’s immigration status and resident areas, higher coverage and timeliness was observed in local residents and affluent areas. For local families who may have higher household incomes [35] and economically developed areas, parents generally had heightened awareness of the importance of EV71 vaccination in the HFMD prevention and were more able to afford cost of EV71 vaccination, leading to higher demand of EV71 vaccination. Besides, the free shift of the migrant population was also a barrier of on-time EV71 vaccination, which was consistent with previous studies [18, 33, 34].

In this study, the urban-rural disparities in EV71 vaccination coverage were observed. The coverage and timeliness of EV71 vaccination was higher in the inner districts than outer districts. This result reflected the urban-rural disparities of the utilization of vaccination services. In recent years, although Chinese governments made great efforts to equalize basic public health services, the distinct unequal utilization of preventive care services between rural and urban still persisted [36]. In Ningbo, inner districts which were urban area of Ningbo had more medical institutions and preventive care services resources than outer districts which were mostly rural area. Shortage of health workforce and unequal distribution of preventive care resources limited vaccination services delivery and usage in rural area largely [32, 36, 37]. In addition, we also noticed that scale of immunization clinics was also associated with the coverage and timeliness of EV71 vaccination. Timeliness of vaccination was highest in large-scaled clinics. Interestingly, our study found lowest timely EV71 vaccination rate in the midsized clinics while the coverage of EV71 vaccine in midsized clinics was even higher than small-sized clinics. In Ningbo, although large-scaled immunization clinics provided vaccination service to more children, they usually located on accessible sites of the core region of the districts and had more health workforce than midsized and small-sized immunization clinics. In fact, the workload of HCWs in the large-scaled immunization clinics were less than those working in other immunization clinics [37], leading to higher coverage and timeliness of vaccination. Instead, midsized immunization clinics often located in suburban region of Ningbo where there is a large migrant population so that the workload of HCWs working there were heaviest [37]. This resulted in the decrease of vaccination timeliness.

Therefore, in order to improve the coverage and timeliness of EV71 vaccination and reduce disparities between urban and rural, resident and migrant, vaccination willingness and actual coverage, more efforts should be made: Firstly, HCWs should recommend the EV71 vaccine to the parents of children under 5 years old as a routine work and provide the information about the effectiveness and safety of EV71 vaccine. Secondly, reminder-recall should be used for the children who may miss the EV71 vaccination. Thirdly, simultaneous administration strategy for EV71 vaccine should be redesigned and add to the current guidelines. Fourthly, health workforce and preventive care services resources should be increased, especially in the rural areas.

This study has several limitations and strengths. First, this study only included children registered in NBCIIMS and those who newly migrated to Ningbo may have been missed. This may resulted in an overestimate of EV71 vaccination coverage in Ningbo. However, we believe the impact is minor, as village doctors work closely with village officials on active searching of new immigrants to make sure that all the children have access to immunization clinics. Second, this study had a narrow time frame because of newly introduction of EV71 vaccine in 2016 in Ningbo. Therefore, we were unable to assess longer trends in EV71 vaccine uptake. Further long term follow-up study is needed. Third, the NBCIIMS data provided limited information about the children and their parents, so we couldn’t explore other determinants of the coverage and timeliness of EV71 vaccination. In the future, more studies are needed to identify motivators and barriers associated with vaccination and to focus on the increase of overall coverage. The strengths of this study is that the large sample size of data obtained from a well-established immunization information system provided high levels of statistical power and internal validity in estimating coverage and utilization patterns of EV71 vaccine.

## Conclusions

Our study showed a low coverage rate and extremely low on-time rate EV71 vaccination in Ningbo since EV71 vaccines were introduced in 2016, yet these rates increased significantly from the 2012 birth cohort to the 2018 birth cohort. Meaningful disparities in coverage and timeliness of EV71 vaccination by children’s immigration status, urbanicity, socioeconomic level of resident areas and scale of immunization clinics were observed. As for utilization patterns, only a few parents chose simultaneous administration of EV71 vaccine and most simultaneous administration occurred at 6–12 months of age with rotavirus vaccine or meningococcal vaccine. Consequently, the finding of our study highlighted the importance of providing relative information about EV71 vaccination and simultaneous administration to improving coverage and timeliness of EV71 vaccination as well as the necessity of eliminating disparities in coverage and timeliness among different populations.

## Data Availability

The datasets used for the current study are not publicly available because they contain detailed vaccination histories of children in Ningbo, but are available from the corresponding author (nbcdcmianyi@163.com) on reasonable request.

## Abbreviations

EV71: Inactivated enterovirus 71
HFMD: Hand, foot and mouth disease
CDC: Center for Disease Prevention and Control
USD: United States dollar
NBCIIMS: Ningbo Childhood Immunization Information Management System
GDP: Gross Domestic Product
OR: Odds Ratio
CI: Confidence Interval
HCW: Health care worker
N/A: Not Applicable
